# Moral Antiseptics

**Published:** 1892-11-26

**Authors:** 


### The Hospital

An Institutional Journal of

Science, Medicine, Nursing, and Philanthropy.

*For Hospitals, Asylums, Infirmaries, Dispensaries, Medical Practitioners, Students, Nurses, and the Charitable Public.*

Vol. XIII.—No. 322.] [Nov. 26, 1892.

### Moral Antiseptics.

Our own generation may claim credit for the scientific demonstration of the principles of antiseptics. The idea of antisepsis is all-pervading; and so, too, are the facts which are its material embodiment. Even the outsider must be impressed by such a fact as this—that one part of corrosive sublimate added to five thousand parts of water will form a solution that will destroy morbid germs, which germs, if undestroyed, have a potency amply sufficient for the destruction of human life. Must there not be a rare virtue in corrosive sublimate!

It will not be denied that if a sublimate for the moral evils which fester in the body politic could be discovered, a signal service would be rendered to all industrious and honest people. Even those who are in love with cynicism, pessimism, and *laissez faire*, do not claim that there is any merit or state virtue in thieving, drunkenness, loafing, and other forms of social parasitism. If we could compound a moral solution which would destroy the germs of moral disease and putrefaction as we can compound a hundred material solutions for the destruction of material putrefaction, even the cynic would be silenced and compelled to keep his worthless cavilling to himself.

It is a profoundly interesting question whether bodily defect or moral depravation is more injurious from the point of view of pure sociology. Is bodily defect the cause of moral depravation? or is moral depravation, when explored to its source, not rather the cause of bodily defect? Or does the truth lie between these two propositions; and is bodily defect sometimes the cause of depravity, and moral depravity sometimes the cause of bodily defect? In whatever way these questions be answered, no man of the least practicality of mind doubts that moral depravity, lying, theft, drunkenness, laziness, and all things of the kind, are utterly bad; injurious to the perpetrator, and quite as injurious to his neighbours, and to the state. It is as imperative upon us to discover and use moral antiseptics as material: many persons whose judgment is of very great weight think it is even more imperative.

A modern doctor was once asked what gift of all which the gods could bestow he would choose for himself if he were offered the choice? "More brains, and better brains," he promptly replied. That is really what we all want! It is a most extraordinary thing that such a vast number of us are so entirely pleased with ourselves and with what we have accomplished in the world, and so inclined to belittle our fellows. What, for example, can be more foolish, and, in practice, more injurious, than for the doctor to regard the parson as a mere consoler of weak-minded women, or for the parson to look upon the doctor as a respectable sort of humbug who would have fewer patients if there were fewer fools in the world? All this kind of vulgar stupidity is worse than child's play; it is only possible to men who have not intellectual capacity enough to take the measure of the world they live in, with its people and its often tragic affairs.

If a doctor wished to offer a justification for dealing occasionally with moral questions he would point straightway to the mountains upon mountains of physical ill and misery which have their roots entirely in moral causes. Perhaps the most wide-reaching of all preventive measures against bodily ills would be the universal purification of the morals of the people. The man who cannot recognise the supreme value of good morals is of the poorest possible mental organisation. At bottom, intellect and morals are one. There cannot be an improvement in morals without a growth in intellect; and, conversely, there cannot be a depravation of morals without an intellectual degradation.

We need, then, our moral sublimate! Is there such an antiseptic to be found? And if there be—what is it? Is education the potent factor, or is art? Again, it is the experienced man who can give us the answer! It is not mere education nor æstheticism, but religion which corresponds in morals to the potent sublimate of which one part in a fluid of five thousand is effectual for purification and health. It is probable that the man who is on the look-out for fresh discoveries will not think we have added much to the stock of things which are scientifically noteworthy by declaring religion to be the most potent of moral antiseptics. He will complain that religion is not new. But neither is the corrosive sublimate which is so powerful a material antiseptic. We were going to say it is as old as religion, and to all intents and purposes it is, and older. But this present generation of scientific medical men is very much in love with sublimate, notwithstanding its want of newness. We do not ask whether it is new or not? We ask whether or not it is an effectual antiseptic, and whether or not it will check the putrefactions which need to be checked, and restore the health which is being destroyed?

An experiment has been carried out in moral antiseptics for a good many years by the Rev. S. A. Barnett, vicar of St. Jude's, Whitechapel. Mr. Barnett is a man of science. In first principles he is entirely at one with Bacon and modern scientists. *Fiat experimentum* in his credo. It is almost the only possible credo nowadays. Mr. Barnett has just published the results of a year's working, exactly as some eminent physician or surgeon might have published the results of treatment in a hundred cases selected for experiment. The antiseptic he has employed has been Christianity as embodied in the life and daily behaviour of cultured and thoroughly convinced persons. Now the essential constituent in this moral antiseptic is intelligent belief. There is no more moral potency in mere professional belief than there is material potency in the box in which the material sublimate may have been packed. It is, we insist, absolutely essential that the Christianity which shall possess moral antisepsis be real and personal, not merely logical and professional.

It is most instructive to note how Mr. Barnett has set to work in the use of his antiseptic. He has used the methods of science; he has, in every department made a point of employing a "salt" which has not "lost its savour." How fundamental this is only scientific persons fully know. The "salt" he has had prepared in solutions of varying strength to suit the needs of the various classes under his spiritual charge. And what classes some of them are in St. Jude's, Whitechapel! Nevertheless, even here, where the atmosphere of things is, if not universally immoral, at least unintellectual and unspiritual, he has destroyed unlimited moral putrefactions, and set up a hundred wholesome and healing centres which, by radiating in all directions, may ultimately lead to the restoration of perfect health in the whole body of St. Jude's parish. A mere list of the agencies called into being for the physical, intellectual, and spiritual purification of Mr. Barnett's parish would fill several lines. The grand fact about them is that they are all well chosen, all of the nature of moral antiseptics, all successful to some worthy extent, and some of them successful in the very highest degree. Let every reader think of this, that the parish of St. Jude's, Whitechapel, is permeated in every part and corner by the quickening impulse of a living, reasonable, and cultured Christianity. Now, is there any man so irrational as not to see what a purification of the moral atmosphere is hereby effected, and what infinite advantage in the prevention of crime and idleness accrues to the body politic?

It is more than unfortunate that the powerful and entirely salutary influence of a man like Mr. Barnett should be limited within the bounds of a parish. The whole of the East-end of London, or, at least, the whole of Whitechapel might with advantage be committed to his charge; and the public, if it were wise, would furnish him liberally with means to multiply his spiritual agencies until they were adequate for the whole district. We make a tremendous and most unscientific mistake in these times of unusual pressure and struggle in undervaluing the policy of moral and spiritual antisepsis. Man is a moral and spiritual being first and above all; and it is not science, but stupidity which refuses to recognise the actual facts, and to deal with humanity exactly as it is.

